# An Imaging and Blood Biomarkers Open Dataset on Alzheimer's Disease vs. Late Onset Bipolar Disorder

**DOI:** 10.3389/fnagi.2020.583212

**Published:** 2020-10-29

**Authors:** Ariadna Besga, Darya Chyzhyk, Manuel Graña, Ana Gonzalez-Pinto

**Affiliations:** ^1^Centre for Biomedical Research Network on MentalHealth, Madrid, Spain; ^2^Department of Internal Medicine, Hospital Universitario de Alava, Vitoria-Gasteiz, Spain; ^3^Computational Intelligence Group, University of the Basque Country (UPV/EHU), San Sebastian, Spain; ^4^ACPySS, San Sebastian, Spain; ^5^Department of Psychiatry, University Hospital of Alava-Santiago, Vitoria-Gasteiz, Spain; ^6^School of Medicine, University of the Basque Country, Vitoria-Gasteiz, Spain

**Keywords:** late onset bipolar disorder, Alzheimer's disease, MRI data, blood biomarkers, machine learning

## 1. Introduction

Bipolar disorder (BD) is a chronic mood disorder alternating maniac/depressive with euthymic episodes. Its onset is conditioned by the environment and genetic inheritance (Bauer M. et al., 2014; Martinez-Cengotitabengoa et al., 2014; Bauer et al., 2015a,b), often during youth producing cognitive, affective, and functional impairment (Forcada et al., 2014). Late onset BD (LOBD) corresponds to ages above 50 years (Depp and Jeste, 2004; Zanetti et al., 2007; Prabhakar and Balon, 2010; Besga et al., 2011; Carlino et al., 2013; Chou et al., 2015). At this period of life it can be difficult to differentiate LOBD from Alzheimer's disease (AD) (Zahodne et al., 2015). Recent studies have found shared biomarkers between LOBD and AD patients (Berridge, 2013). Similar roles of inflammation and oxidative stress biomarkers have been found in AD (Akiyama et al., 2000; Kamer et al., 2008; Sardi et al., 2011), LOBD (Goldstein et al., 2009; Konradi et al., 2012; Leboyer et al., 2012; Lee et al., 2013; Bauer I et al., 2014; Hope et al., 2015), depression, and mania (Brydon et al., 2009; Dickerson et al., 2013; Castanon et al., 2014; Singhal et al., 2014). Further details of shared LOBD and AD traits are given in Besga et al. (2015b). Specific common psychiatric symptoms are: agitation, euphoria, disinhibition, over-activity without agitation, aggression, affective liability, dysphoria, apathy, impaired self-regulation, and psychosis (Albert and Blacker, 2006; Zahodne et al., 2015). The demographic, neuropsychological, clinical, imaging, and blood plasma analytics data used in our study, as well as the rules for eligibility and discarding of patients, appear in Graña et al. (2011) and Besga et al. (2012, 2015a, 2016, 2017), hence reproducing them here would be self-plagiarism according to journal rules. The content of this paper is as follows: first we refer the contents of the dataset and its location for downloading, then we summarize previously published results. We include the ethics statement and the trial registration reference.

## 2. Contents of the Dataset

The dataset has been published in the Zenodo public repository (Besga et al., 2020). Its contents are as follows:

- The clinical data includes the following information
  - Demographics data, such as age, sex, civil state, environment conditions, and others
  - Results of neuropsychological examination: we have carried out tests for executive function, learning and memory, and attention. Details are given in Besga et al. (2015a).
  - Clinical observation: we carried out the Neuropsychiatric Inventory and the functional assessment staging (FAST). Details are given in Besga et al. (2015a).
  - Blood plasma biomarkers: Neurotrophins, inflammation biomarkers, and oxidative stress biomarkers. Details are given in Besga et al. (2017).
- Magnetic Resonance Imaging (MRI) data obtained on a 1.5 Tesla scanner, that includes the following (details of data capture and preprocessing are given in Graña et al., 2011; Besga et al., 2012, 2016):
  - Diffusion weighted imaging (DWI) with high b and 30 gradient directions. The original data is provided in the dataset.
  - Fractional Anisotropy (FA) volumes computed from the DWI data after noise correction, registration, and diffusion tensor (DTI) computation. The original FA data and the FA volumes co-registered to MNI template using the T1-weighted data non-linear registration parameters are provided.
  - T1-weighted anatomical volumes at 1 mm resolution. Both original and non-linearly registered to MNI template data are provided in the published dataset.
- Results: we provide the following results that can be used as reference for checking the integrity of the data
  - Voxel based morphometry (VBM) computed using Statistical Parametric Mapping (SPM) software.^1^
  - Tract based spatial statistics (TBSS) intermediate and final results, as well as the correlations with blood sample biomarkers computed using the FSL software (Smith et al., 2004)^2^ as published in Besga et al. (2017).

## 3. Published Results

Previously reported results of this study are as follows:

1. Machine learning based computer aided diagnosis (CAD) (Sigut et al., 2007; Salas-Gonzalez et al., 2009; Ramirez et al., 2010; Savio et al., 2011; Westman et al., 2011; Termenon et al., 2013) achieved high accuracy discrimination between AD and LOBD populations using whole brain FA (Graña et al., 2011; Besga et al., 2012). We discuss there that the good classification performance was not enough due to poor brain localization results causes by the feature extraction process.
2. Again, machine learning techniques applied on the clinical, neuropsychological test, and blood plasma biomarkers give good LOBD vs. AD classification performance (Besga et al., 2015a). Clinical variables reported the highest discriminant power, while blood plasma biomarkers reported low discriminant power. Combinations of both, improved the classification results, showing some indirect effects of blood plasma biomarkers.
3. Using eigenanatomy tools (Avants et al., 2012, 2014) we found an optimal decomposition of the FA volumes that show maximal correlation with plasma biomarkers (Besga et al., 2016). This decomposition provides the features for classifier model building to discriminate AD vs. LOBD classification, providing anatomical localization of the effects corresponding to the classification features, which are consistent with differential diagnostics.
4. The Tract-Based Spatial Statistics (TBSS) (Smith et al., 2006; Bach et al., 2014) allows us to identify strongly significant clusters in behavioral impairment relevant tracts. These clusters show specific correlation with neurotrophins biomarkers in an AD population but none with a LOBD population. We also found a strong positive correlation of inflammation biomarkers with the LOBD population (Besga et al., 2017).

## 4. Limitations

The major limitation for the data is the small sample size. Recruiting was a long process that took several years due to the advanced age of the participants. Another limitation to keep in mind is that the plasma biomarkers were not extracted specifically from the central nervous system (CNS) tissues, so they are biomarkers of the general state of the body not of specific locations in the CNS.

## 5. Conclusions

We introduce a public dataset that can be exploited for the identification of biomarkers that allow enhanced differential diagnosis of AD vs. LOBD. We summarize our findings, already published in the literature, in order to encourage innovative computational approaches to be tested on this dataset.

## Data Availability Statement

The datasets presented in this study can be found in online repositories. The names of the repository/repositories and accession number(s) can be found at: Zenodo, doi: 10.5281/zenodo.3935636.

## Ethics Statement

The ethics committee of the Alava University Hospital, Spain, approved this study. All patients provided written consent to participate in the study, which was conducted according to the provisions of the Helsinki declaration. The study has been registered as an observation trial^3^ in the ISRCTN registry.

## Author Contributions

AB and AG-P have made a substantial contributions to the conception and design of the work. DC and MG contributed to the acquisition, analysis, and interpretation of data for the work. All authors contributed to drafting the work and revising it critically for important intellectual content, provided the final approval of the version to be published, and agree to be accountable for all aspects of the work in ensuring that questions related to the accuracy or integrity of any part of the work are appropriately investigated and resolved.

## Conflict of Interest

The authors declare that the research was conducted in the absence of any commercial or financial relationships that could be construed as a potential conflict of interest. The reviewer FS declared a past co-authorship with one of the authors MG to the handling Editor.

## Footnotes

^1^https://www.fil.ion.ucl.ac.uk/spm/

^2^http://fsl.fmrib.ox.ac.uk/fsl/fslwiki/TBSS

^3^http://www.controlled-trials.com/search?q=HS%2FPI2010001
